# Sustained Release of Prindopril Erbumine from Its Chitosan-Coated Magnetic Nanoparticles for Biomedical Applications

**DOI:** 10.3390/ijms141223639

**Published:** 2013-12-03

**Authors:** Dena Dorniani, Mohd Zobir Bin Hussein, Aminu Umar Kura, Sharida Fakurazi, Abdul Halim Shaari, Zalinah Ahmad

**Affiliations:** 1 Materials Synthesis and Characterization Laboratory (MSCL), Institute of Advanced Technology (ITMA), Universiti Putra Malaysia, Selangor 43400, Malaysia; E-Mail: dena_dorniani@yahoo.com; 2 Vaccines and Immunotherapeutics Laboratory (IBS), Universiti Putra Malaysia, Selangor 43400, Malaysia; E-Mails: aminuukura@yahoo.com (A.U.K.); sharida@upm.edu.my (S.F.); 3 Department of Physics, Faculty of Science, Universiti Putra Malaysia, Selangor 43400, Malaysia; E-Mail: ahalim@upm.edu.my; 4 Chemical Pathology Unit, Department of pathology, Faculty of Medicine and Health Sciences, Universiti Putra Malaysia, Selangor 43400, Malaysia; E-Mail: zalinah@upm.edu.my

**Keywords:** superparamagnetic nanoparticles, chitosan, prindopril erbumine, drug delivery

## Abstract

The preparation of magnetic nanoparticles coated with chitosan-prindopril erbumine was accomplished and confirmed by X-ray diffraction, TEM, magnetic measurements, thermal analysis and infrared spectroscopic studies. X-ray diffraction and TEM results demonstrated that the magnetic nanoparticles were pure iron oxide phase, having a spherical shape with a mean diameter of 6 nm, compared to 15 nm after coating with chitosan-prindopril erbumine (FCPE). Fourier transform infrared spectroscopy study shows that the coating of iron oxide nanoparticles takes place due to the presence of some bands that were emerging after the coating process, which belong to the prindopril erbumine (PE). The thermal stability of the PE in an FCPE nanocomposite was remarkably enhanced. The release study showed that around 89% of PE could be released within about 93 hours by a phosphate buffer solution at pH 7.4, which was found to be of sustained manner governed by first order kinetic. Compared to the control (untreated), cell viability study in 3T3 cells at 72 h post exposure to both the nanoparticles and the pure drug was found to be sustained above 80% using different doses.

## 1. Introduction

Drug nanoparticles are a drug delivery system with the ability to load, carry and release different types of drugs particularly on target cells. Lately, nanoparticles received considerable attention in biological applications [1,2]. Unfortunately, due to the strong magnetic dipole-dipole attractions between particles, iron oxide nanoparticles tend to agglomerate. Polymers, including crosslinked dextran [3,4], poly(ethylene glycol) [5–7], poly(lactic acid) [8], poly(ethylene imine) (PEI) [9], polyvinyl alcohol [7,10] and chitosan [11] are widely used to amend the stability and biocompatibility of the superparamagnetic iron oxide nanoparticles [12–14]. The lack of colloidal stability of the PEI polymer is the biggest problem in relation to the cationic polymer PEI coated with iron oxide nanoparticles [7].

Chitosan is a cationic, hydrophilic and partially acetylated glucosamine polymer with many useful properties, such as hydrophilicity, low immunogenicity, low toxicity, biocompatibility, and high biodegradability [14,15]. The primary amine groups give special properties to chitosan and make it appropriate for further functionalization with specific components, such as various drugs, specific binding sites, or other functional groups [16]. Therefore, the cationic nature of chitosan permits for ionic cross linking with multivalent anions, and could be a suitable kind of polymer that can be used to modify the iron oxide nanoparticles. It is required that the magnetite nanoparticles possess high saturation magnetization for biomedical applications such as magnetic resonance imaging (MRI) and hyperthermia [14].

According to the description set forth in the sixth report of the Joint National Committee on Prevention, Detection, Evaluation and Treatment of High Blood Pressure, almost 25% of US adults suffering of hypertension [17,18]. Prindopril, is a tert-butylamine salt of 1-{(2S)-2-[(1S)-1-carbethoxybutyl)amino]-1-oxopropyl}-(2S,3As,7As)-perhydroindole-2-carboxilic acid, belongs to the class of antihypertensive drugs, a long acting angiotensin-converting enzyme (ACE) inhibitor, which is impressive in the treatment and prevention of various medical conditions such as med of mild-to-moderate hypertension [19], congestive heart failure, diabetic nephropathy and post-myocardial infarction [20–23]. It is an acid-ester prodrug, which is deesterified by esterases in the liver and after oral administration, and it is converted to the active diacid perindoprilat by hydrolysis [24].

Due to the lack of studies on perindopril erbumine (PE) as a model drug, we selected this to be coated on the surface of iron oxide nanoparticles and chitosan as a formulation of the drug delivery. Moreover, the main objective of this work was to focus on improving the sustained release properties of the as-synthesized nanocomposite, due to the fast release that was observed in Zn/Al-LDH intercalated with PE [25].

## 2. Results and Discussion

### 2.1. Powder X-ray Diffraction

**Figure 1.**
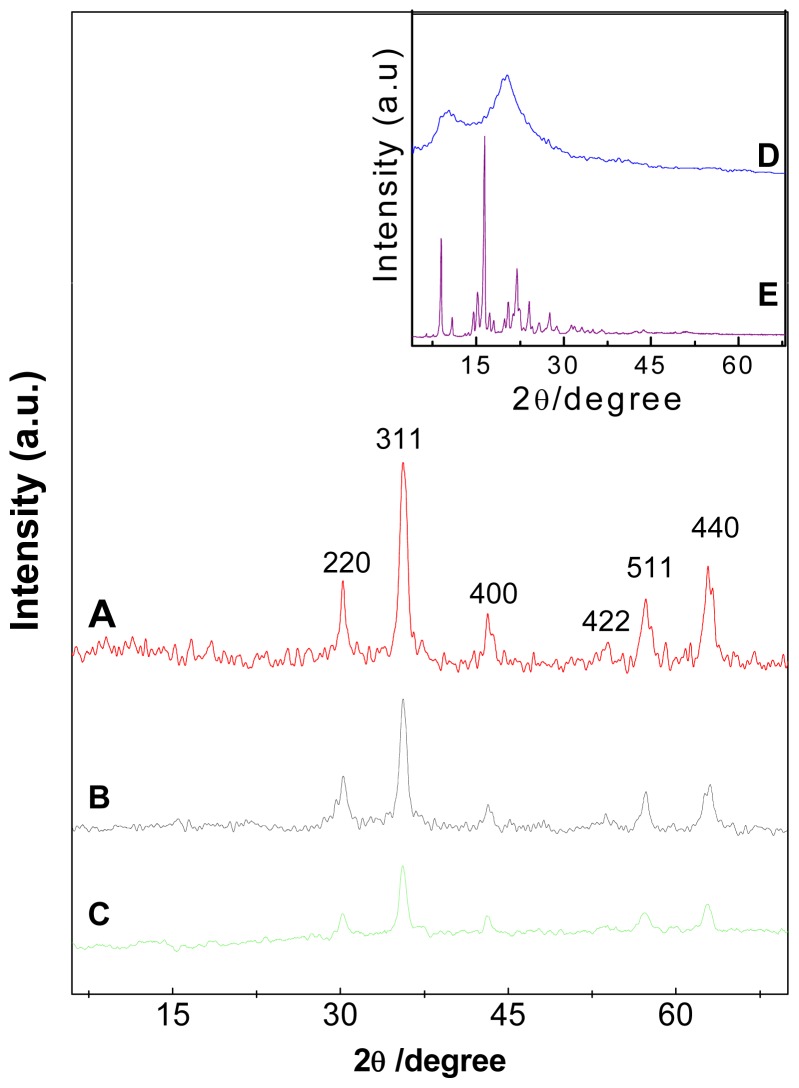
XRD patterns of Fe_3_O_4_ nanoparticles (**A**) iron oxide coated with chitosan (FC) (**B**); and iron oxide coated with chitosan-prindopril erbumine (FCPE) (**C**); Inset shows the X-ray diffraction patterns for the chitosan (**D**) and pure perindopril erbumine (PE) (**E**).

Powder X-ray diffraction patterns of pristine naked Fe_3_O_4_ nanoparticles, Fe_3_O_4_ nanoparticles coated with chitosan (FC) and iron oxide nanoparticles coated with chitosan-perindopril erbumine (FCPE) are depicted in Figure 1A–C, respectively. Figure 1D (pure chitosan) shows two main diffraction peaks at 2θ = 10.5° and 20.6° [26,27]. As shown in Figure 1A, a series of characteristic peaks of iron oxide (2θ = 30.2°, 35.7°, 43.3°, 53.9°, 63.2°) can be assigned as (220), (311), (400), (511) and (440), respectively. These peaks are consistent with the standard pattern (JCPDS No. 19-629), which shows that the magnetite were pure iron oxide with a cubic inverse spinal structure. In addition, these peaks were observed in FCPE (Figure 1C); this indicates that the modification procedure did not change the crystal structure of iron oxide nanoparticles. The characteristic peaks of perindopril erbumine shown in Figure 1E were absent from the X-ray diffraction pattern of the product (FCPE), suggesting that perindopril erbumine was coated on iron oxide together with chitosan as the matrix. The mean crystallite size, *D* of magnetite iron oxide nanoparticles was calculated by the Debye-Scherrer equation (*D* = Kλ/βcosθ) [14,28] where, K is a constant (K = 0.9 for Cu-K_α_), λ is wavelength (0.15406 nm), β is the peak width of half-maximum and θ is the diffraction angle. The particle size of pure iron oxide thus obtained from this formula was about 4 nm.

### 2.2. Fourier Transforms Infrared Spectroscopy

**Figure 2.**
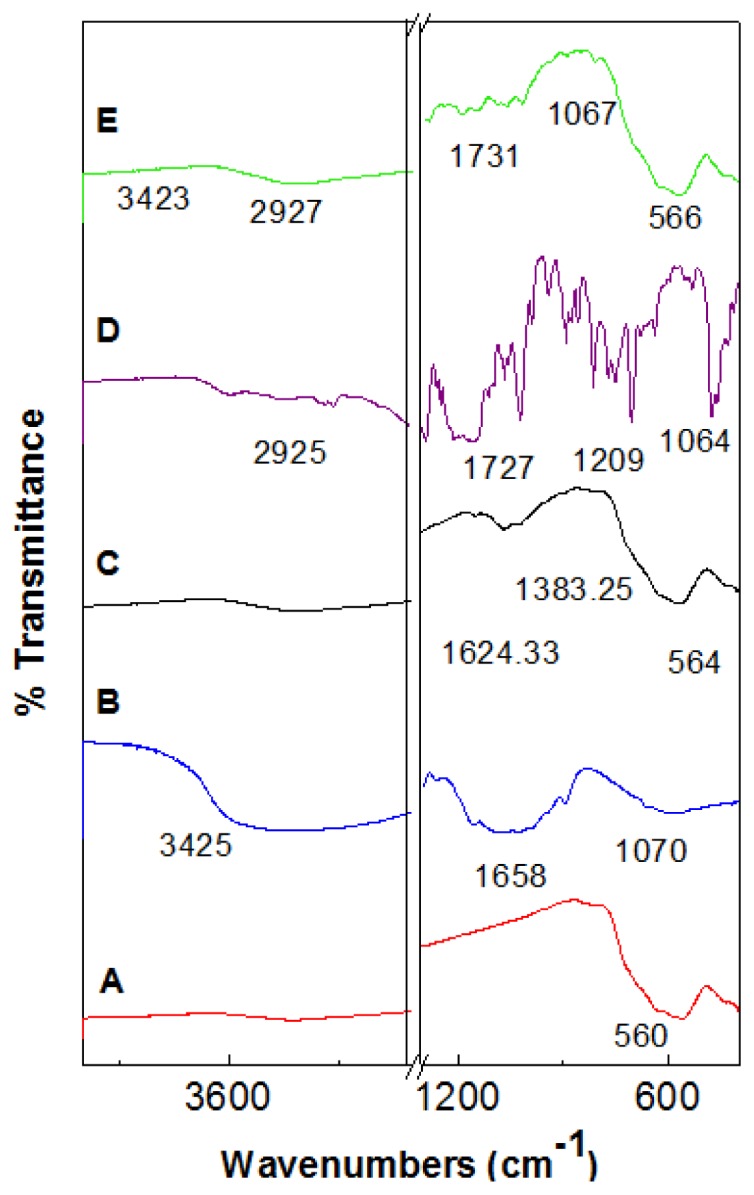
FTIR spectra of (**A**) iron oxide nanoparticles; (**B**) chitosan; (**C**) iron oxide nanoparticles coated with chitosan (FC); (**D**) prindopril erbumine (PE) and (**E**) iron oxide nanoparticles coated with chitosan-prindopril erbumine (FCPE).

Figure 2 show the FTIR spectrum of pristine Fe_3_O_4_ nanoparticles (A), pure chitosan (B), iron oxide nanoparticles coated with chitosan (C), perindopril erbumine (D) and Fe_3_O_4_ nanoparticles coated with chitosan and perindopril erbumine (E). The peak at around 560 cm^−1^ observed in Figure 2A, relates to Fe–O–F bands in Fe_3_O_4_ nanoparticles. However, this characteristic peak of Fe–O–F shifts to 566 cm^−1^ after coating procedure (Figure 2E). The characteristic bands of pure chitosan can be observed at around 3425 cm^−1^ (O–H stretching and N–H stretching vibrations), 1658 cm^−1^ (amide) and 1064 cm^−1^ (C–O–C stretching vibration) [29]. The characteristic peak observed at 1624 and 1383 cm^−1^, can be assigned to N–H bending vibration and –C–O stretching of the primary alcoholic group in chitosan, respectively (Figure 2C).

As can be observed in the FTIR spectra of perindopril erbumine (Figure 2D) there are many intense, sharp absorption peaks that are due to different functional groups present in molecules such as primary amine, secondary amine, ester, carboxylic acid and methyl groups. The peak at 1247 cm^−1^ is due to C_3_C–N stretching [30] and 1022 cm^−1^ is due to C–N stretching [31]. The band at 2925 cm^−1^ indicates that CH in NH–CH-propyl is shifted to 2927 cm^−1^ after coating procedure (Figure 2E). The secondary amine functional group recorded a peak at 1154 cm^−1^ which is due to the symmetric stretching of C–N–C. The band at 1727 cm^−1^ and 1064 cm^−1^ in Figure 2D are due to C=O and O–C–C stretching in the ester group, respectively and shifted to 1731 cm^−1^ and 1067 cm^−1^ in FCPE (Figure 2E). The band recorded at 1291 cm^−1^ is due to C–O stretching in the carboxylic acid group (Figure 2D) [32].

The broad peak at 3423 cm^−1^ which is observed in Figure 2E can be assigned to O–H stretching [33]. FTIR spectra of FCPE compound confirming the existence of chitosan and perindopril erbumine which were successfully coated on the surface of iron oxide.

### 2.3. Magnetic Properties

**Figure 3.**
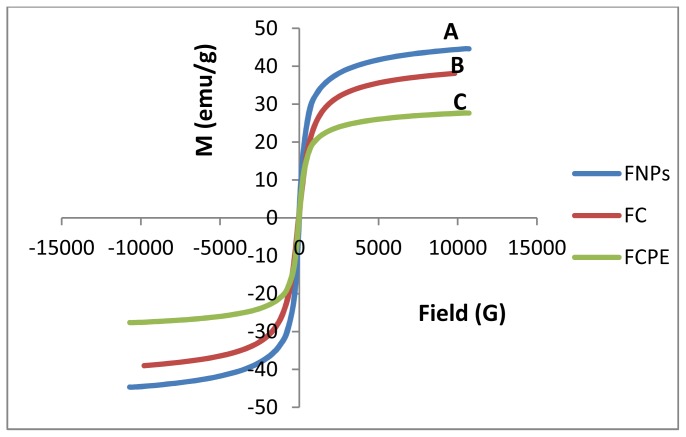
Magnetization curves of (**A**) iron oxide magnetic nanoparticles (FNPs), (**B**) iron oxide coated with chitosan (FC) and (**C**) iron oxide nanoparticles coated with chitosan-prindopril erbumine (FCPE).

Hysteresis loops of Fe_3_O_4_ nanoparticles, FC (iron oxide coated with chitosan) and FCPE (iron oxide nanoparticles coated with chitosan-perindopril erbumine), were characterized by a vibrating sample magnetometer (VSM) as a function of the magnetic field at room temperature (Figure 3). The values of saturation magnetization (*M*_s_), remanent magnetization (*M*_r_) and high coercivity (*H*_ci_) are shown in Table 1. The saturation magnetization of magnetic iron oxide nanoparticles was about 44.65 emu/g compared to 38.57 emu/g after coated with chitosan and 27.66 emu/g, for the iron oxide coated with chitosan-PE. The decrease of saturation magnetization could be due to the existence of polymer on the surface of iron oxide nanoparticles, which changes the surface magnetic anisotropy and leads to enhancement of the surface spins disorientation [34]. According to the VSM results, it was proved that all samples showed superparamagnetic behavior, *i.e.*, after removal of the external magnetic field they did not retain any magnetism. Therefore, due to the high magnetization and superparamagnetic properties of FCPE, it can be used in biomedical applications.

### 2.4. Thermal Analyses

**Figure 4.**
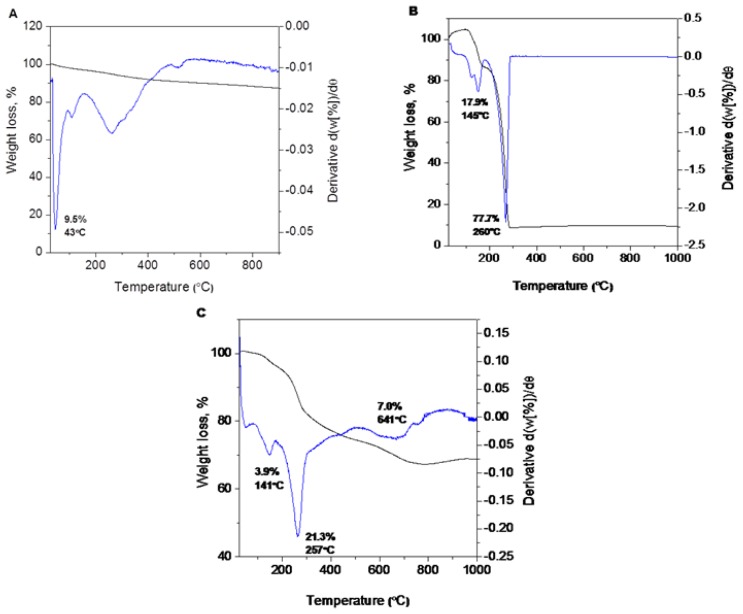
TGA of (**A**) iron oxide nanoparticles; (**B**) perindopril erbumine; and (**C**) iron oxide nanoparticles coated with chitosan-perindopril erbumine (FCPE).

The thermal behavior of the pure drug (PE) and magnetic iron oxide nanoparticles before and after coating with chitosan-perindopril erbumine (FCPE) was measured using thermogravimetric and differential thermogravimetric analyses (Figure 4). The total weight loss over the temperature range from 25 to 900 °C for uncoated nanoparticles is about 9.5% that can be explained by the loss of residual water in the sample (Figure 4A) [35]. The differential thermogravimetric curve shows two main thermal events. The first one which corresponding to a sharp peak occurred at 145 °C with 17.9% weight loss (Figure 4B) may attribute to melting of perindopril erbumine. The second stage of mass loss at 260 °C with 77.7% mass reduction is attributed to the decomposition and subtle combustion of perindopril erbumine [36].

Figure 4C shows that the decomposition of FCPE progressed through three major stages of weight loss, occurring at temperature maxima of 141 °C, 257 °C and 641 °C with weight losses of 3.9%, 21.3% and 7.0%, respectively. The first stage of mass loss at 141 °C is due to the removal of water physisorbed on the external surface of nanoparticles as well as structured water. The sharp second mass reduction at 257 °C might be due to the decomposition of chitosan. The temperature region in FCPE is clearly higher than the pure free perindopril erbumine, which suggests that the thermal stability of perindopril erbumine in nanoparticles was enhanced due to the coating process which involves electrostatic attraction between the iron oxide surface, chitosan and perindopril erbumine.

### 2.5. Determination of Average Size and Size Distribution Properties

**Figure 5.**
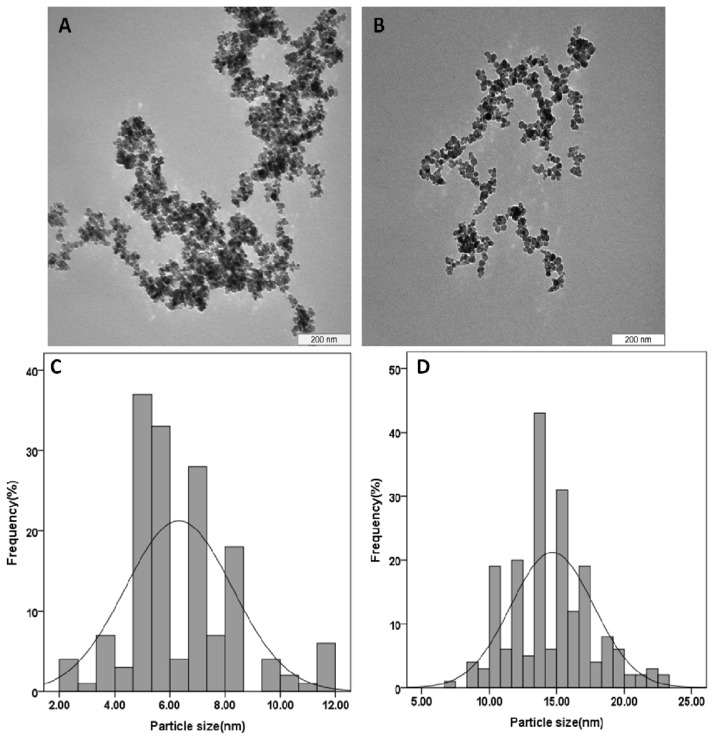
TEM micrographs (**A**) iron oxide magnetic nanoparticles with 200 nm microbar; (**B**) iron oxide nanoparticles coated with chitosan-prindopril erbumine (FCPE) with 200 nm microbar; (**C**) particle size distribution of iron oxide nanoparticles; and (**D**) particle size distribution of FCPE nanocomposite.

The typical TEM images and particle size distribution of the pristine Fe_3_O_4_ and its coated by chitosan-perindopril erbumine (FCPE) are shown in Figure 5. The particles with nanometer size were successfully prepared by co-precipitation method. The particle size and size distribution of the particles were determined by measuring at least 200 nanoparticles randomly using image analysis software (Figure 5C,D). It was clear that the as prepared iron oxide nanoparticles and FCPE nanocomposite display roughly spherical shapes (Figure 5A,B). The mean diameter of naked iron oxide nanoparticles is about 6 ± 2 nm, whereas the average size of FCPE nanocomposite is around 15 ± 3 nm (Figure 5C,D), in agreement with the XRD results. The increase of the size in FCPE nanocomposite can be proved due to the formation of the iron oxide which is coated with chitosan and perindopril erbumine with “core-shelled structure” [5].

### 2.6. Loading and Release Behavior of Perindopril Erbumine

**Figure 6.**
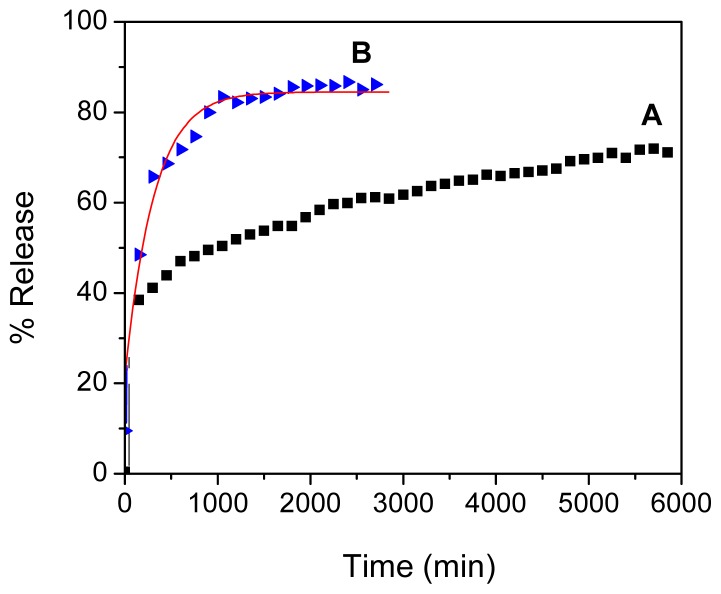
Release profiles of prindopril erbumine (PE) from the iron oxide nanoparticles coated with chitosan-prindopril erbumine (FCPE) into phosphate buffered solution at pH 7.4 (**A**) and pH 4.8 (**B**).

Using an UV-visible instrument and calibration curve equation, the loading of perindopril erbumine in FCPE nanocomposite was estimated to be 62%. From previous work, it is obvious that the release process is slower and more stable at pH 7.4 compared to pH 4.8, due to an anion exchange process between perindopril erbumine and buffer solution [37,38]. The release profiles of PE from the FCPE nanocomposite were measured in an aqueous solution of phosphate-buffered solution at pH 7.4 and 4.8 (Figure 6A,B). The release profiles show that the maximum percentage release of PE from FCPE reaches about 72.2% within about 5631 min (94 h) at pH 7.4 compared to 85.8% within about 2743 min (46 h) when exposed to a solution at pH 4.8. Compared to previous studies, the release behavior of peridopril erbumine, which was loaded in FCPE, is more stable and slower than the one in which layered double hydroxides were used as the matrix [25].

### 2.7. Release Kinetics of Perindopril Erbumine from the Nanocomposite

In order to further study the release behavior of perindopril erbumine from FCPE nanocomposite, first-order (Equation 1), pseudo-second order (Equation 2) and parabolic diffusion (Equation 3) models were chosen [39–42].

(1) $$\text{ln }(q_{\text{e}}-q_{t})=\text{ln }q_{\text{e}}-k_{1}t$$

(2) $$t/q_{t}=1/k_{2}{q_{\text{e}}}^{2}+t/q_{\text{e}}$$

(3) $$(1-M_{t}/M_{0})/t=kt^{-0.5}+b$$

Where, *k* is the corresponding release amount constant, *M*_0_ and *M_t_* are the drug content remained in FCPE nanocomposite at release time 0 and *t*, respectively, *q*_e_ and *q_t_* are the equilibrium release amount and the release amount at time *t*, respectively.

**Figure 7.**
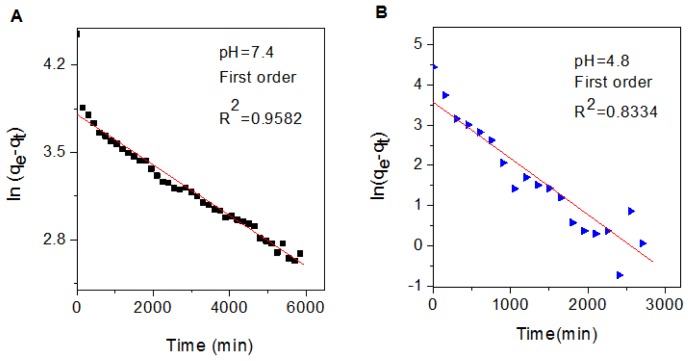
Kinetics of controlled release by fitting the data of perindopril erbumine release from iron oxide nanoparticles coated with chitosan-prindopril erbumine (FCPE) into phosphate-buffered solutions at pH 7.4 (**A**) and pH 4.8 (**B**) using first-order kinetics model.

Using these three kinetic models as mentioned earlier in fitting the release kinetic data, it was found that the first-order kinetic model is more satisfactory to describe the release kinetic process of perindopril erbumine from the FCPE nanocomposite compared to other models used in this study. Figure 7 shows plot of the fitting of prindopril erbumine released from FCPE nanocomposite. At pH 7.4, the correlation coefficient (*R*^2^) and *k* values are 0.9582 and 2.02 × 10^−4^ mg/min, respectively compared to 0.8334 and 1.4 × 10^−3^ mg/min for pH 4.8, respectively. The resulting correlation coefficient (*R^2^*), percentage of saturation release, rate constant (*k*) and half time (*t*_1/2_) is given in Table 2.

### 2.8. *In Vitro* Bioassay

**Figure 8.**
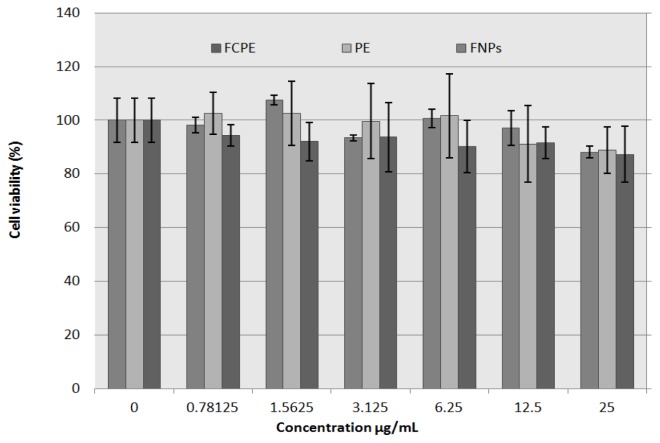
Cell viability assay using prindopril erbumine (PE), iron oxide nanoparticles (FNPs) and iron oxide nanoparticles coated with chitosan-prindopril erbumine (FCPE) on normal fibroblast (3T3 cells) 72 h post exposure.

Cell viability was sustained with an increase in doses, and no significant cell death (cytotoxicity) was observed with all the three agents exposures within the dose range of 0.78–25 μg/mL compared to the control (untreated wells). More than 80% of the exposed cells survived when incubated with up to 25 μg/mL of iron oxide nanoparticles (FNPs), pure drug (PE) and FCPE nanocomposite, as used in this experiment (Figure 8). Previous work on the perindopril erbumine intercalated into layered double hydroxide was found to have no cytotoxic effect on normal Chang liver cells, 24 h after exposure [25]. In the related study, in which iron oxide nanoparticles exposed to both normal and cancerous cells, the viability was observed to be affected at doses above or equal 100 μg/mL, below which cell viability was generally found to be above 80% [43].

Cell viability study of 3T3 cell line, 72 h after exposure using prindopril erbumine (PE), iron oxide nanoparticles (FNPs) and FCPE nanocomposite on normal fibroblast (3T3 cells) was investigated. The figure shows a slight decrease in cell viability, which is dose-dependent, with more than 80% cell survival at the highest concentration used in the entire three compounds. There is no significant difference between the three groups with respect to viability, base on concentration used with *p* value of 0.134 (*p* > 0.05).

## 3. Experimental Section

### 3.1. Materials and Methods

Low molecular weight chitosan (75%–85% degree of deacetylation) was purchased from Sigma-Aldrich (St. Louis, MO, USA). Perindopril erbumine (C_23_H_43_N_3_O_5_, with molecular weight 441.6 g/mol) was purchased from CCM Duopharma (Klang, Malaysia) at 99.79% purity and used as received. Iron chloride tetrahydrate (FeCl_2_·4H_2_O ≥ 99%) and iron chloride hexahydrate (FeCl_3_·6H_2_O, 99%) were purchased from Merck KGaA (Darmstadt, Germany). Acetic acid solution 99.8% was used as a solvent of chitosan, purchased from Hamburg Industries Inc, Hamburg, Germany. Distilled deionized water was used to prepare all aqueous solutions (18.2 M·Ω·cm^−1^).

### 3.2. Synthesis of Magnetic Nanoparticles and Coating Procedure

Superparamagnetic iron oxide nanoparticles were prepared as previously reported by Lee and co-workers [44]. The mixture of 2.43 g ferrous chloride tetrahydrate, 0.99 g ferric chloride hexahydrate, 80 mL deionized water and 6 mL ammonia hydroxide (25% by mass) was exposed to ultrasonic irradiation for 1 h. After the precipitates were centrifuged for three times and dispersed in 100 mL deionized water, the mixture of chitosan-PE with the ratio of 2:2 was added to the solution and the mixture was stirred for 24 h. Finally, the precipitate was collected by a permanent magnet and then dried in an oven. Because of the affinity toward the surface of iron oxide (negative charge) and the amine groups of chitosan (positive charge), chitosan was coated on the surface of the nanoparticles through physical absorption. It was previously reported that the unbound primary amino groups, hydrogen bonding and electrostatic attraction play an important role in preventing the aggregation of chitosan which is coated with superparamgnetic iron oxide nanoparticles [14].

### 3.3. Cell Culture

The 3T3 cell, purchased from American Tissue culture center (ATCC, Manassas, VA, USA) was cultured in RPMI 1640 medium (Sigma-Aldrich, St. Louis, MO, USA) supplemented with 10% fetal bovine serum (Invitrogen Corp., Auckland, New Zealand), 1% antibiotics comprising 100 units/mL penicillin and 100 μg/mL streptomycin. Cells were incubated at 37 °C in humidified 5% CO_2_/95% air and used for seeding and treatment at 90% confluent. Enzymatically, the confluent cell layers were removed, using Trypsin/EDTA (Gibco, Big Cabin, OH, USA), and re-suspended in new culture medium. The MTT assay and cell viability study was performed in order to observe the toxic effect of iron oxide nanoparticles coated with chitosan and perindopril erbumine on normal cells.

### 3.4. Cytotoxicity Study

3T3 (normal fibroblast) cells were seeded at a density of 0.5 × 10^4^ cells/well into 96-well plates and kept at 5% CO_2_ and 37 °C for 24 h, to allow for cells to attach. Stock solution of iron oxide nanoparticles (FNPs), pure drug prindopril erbumine (PE) and FCPE nanocomposite were individually suspended in the culture medium at concentration of 10 mg/mL and dispersed by ultrasonic vibration for 30 min, subsequently diluted in complete media to a desired concentration, through a serial dilution dose range of 0.78–25 μg/mL was used for treatment and 0 μg/mL was used as control. To achieve a desired uniform suspension, each concentration was stirred on vortex agitator (2 min) before use. Seventy two hours post exposure viability assay was done to asses’ toxicity of the three agents compared to control. Cell viability was determined using a colorimetric assay based on the MTT solution conversion to soluble formazin by viable cells. In brief, 20 μL of MTT solution (5 mg/mL of PBS) was added to each well and kept in an incubator for 3 h. The MTT-containing medium was removed gently and replaced with dimethyl sulfoxide (200 μL/well) to mix the formazan crystals until dissolved. Absorbance at 570 and 630 nm (background) was measured with a micro-plate Elisa reader (ELx800 from BioTek Instruments, Winooski, VT, USA). All experiments were carried out in triplicate.

### 3.5. Drug Releasing Procedure

Drug release profiles of perindopril erbumine from FCPE were obtained at room temperature using phosphate-buffered solution at pH 7.4 and 4.8 [25,39,45]. The PE released was achieved by adding 6 mg FCPE nanocomposite into the mixture of 1 mL HCl and 3 mL HNO_3_ and marked it up to 25 mL by deionized water and stirred for 24 h. The accumulated amount of prindopril erbumine released into the solutions was measured at different times at 215 nm.

### 3.6. Characterization

X-ray powder diffraction patterns were recorded in the range of 6°–70° to determine the crystal structure of samples on an XRD-6000 (Shimadzu, Tokyo, Japan), using Cu-K_α_ radiation (λ = 1.5406 Å) at 30 kV and 30 mA, with a dwell time of 0.5 degrees per minute. Fourier transform infrared spectra (FTIR) were recorded over the range of 400–4000 cm^−1^ on a Thermo Nicolet Nexus FTIR (model Smart Orbit, Madison, WI, USA) with 4 cm^−1^ resolution, using the KBr disc method to analyze the interaction between iron oxide, chitosan and perindopril erbumine. Thermogravimetric and differential thermogravimetric analyses were obtained using a Mettler-Toledo TGA/SDTA 85^e^ instrument (Greifensee, Switzerland) with a heating rate of 10 °C/minute, in the range of 20–1000 °C in a 150 μL alumina crucibles under a nitrogen atmosphere. A transmission electron microscopy (Hitachi H-7100, Tokyo, Japan) was used to observe the morphology and particle size of nanoparticles at an accelerating voltage of 100 kV. A Shimadzu 1650 series UV-vis spectrophotometer (Shimadzu, Tokyo, Japan) were used to determine the drug release from the nanocomposite.

## 4. Conclusions

A new drug nanocarrier was synthesized by coating chitosan and prindopril erbumine onto the surface of iron oxide nanoparticles using a simple coating method. X-ray diffraction patterns and TEM results showed that the magnetic nanoparticles were pure iron oxide with a cubic inverse spinal structure with an average diameter of 6 nm, compared to 15 nm after the coating process. It is apparent that prindopril erbumine was released in a controlled manner with around 89% within about 93 h by phosphate-buffered solution at pH 7.4 and governed by first-order kinetics. Prindopri erbumine, iron oxide nanoparticles and its coated nanocomposite, FCPE were not toxic in a normal human fibroblast (3T3) cell line. Therefore, our nanocomposite containing prindopril erbumine is a possible alternative drug delivery method with minimal toxicity potential.

## Figures and Tables

**Table 1.** Magnetic properties of iron oxide nanoparticles and iron oxide coated with chitosan-prindopril erbumine (FCPE).

Sample	*M*_s_ (emu/g)	*M*_r_ (emu/g)	*H*_ci_ (G)
Fe_3_O_4_	44.655	1.5714	21.955
FC	38.573	0.6971	24.065
FCPE	27.664	1.1869	27.002

**Table 2.** Correlation coefficient, rate constant, and half-time obtained by fitting the release data of prindopril erbumine (PE) from iron oxide nanoparticles coated with chitosan-prindopril erbumine (FCPE) into phosphate-buffered solution at pH 7.4 and 4.8.

Aqueous Solution	Saturated Release (%)	*R*^2^			Rate constant (k) a (mg/min)	*t*_1/2_^a^ (min)

		First-order	Pseudo-second order	Parabolic diffusion		
pH 7.4	85.8	0.9582	0.4776	0.9054	2.02 × 10^−4^	3431
pH 4.8	72.2	0.8334	0.6760	0.7367	1.4 × 10^−3^	495

a Estimated using first order kinetics.
